# Observations on the Properties Commonly Attributed by Medical Writers to Human Milk, on the Changes It Undergoes in Digestion, and the Diseases Supposed to Originate from This Source in Infancy

**Published:** 1790

**Authors:** Joseph Clarke


IX. *Obfervations on the Properties commonly attributed by medical Writers to Human Milk, on the Changes it undergoes in Digeftion, and the Difeafes fuppofed to originate from this Source in Infancy.* *By* Jofeph Clarke, *M.D. M.R.I.A.* — *From The Tranfactions of the Royal Irifh Academy, for the Year* 1788.

SOME years ago, when I was appointed affiftant to the Lying-in Hofpital of this city, an uncommon mortality prevailed among the infants born therein. Induced by this difagreeable neceffity to perufe the works of many of the principal medical writers relating to infantile difeafes, I was forcibly ftruck with the fimplicity and uniformity of their pathology on on the ſubject. For more than a century paſt it has been very generally ſuppoſed that the diſeaſes of infants are all of the ſame genus, proceed from the ſame cauſes, and differ only in degrees *. Natural ſenſibility and delicacy of frame have been conſidered as the prediſponent cauſe, and predominant acidity in the ſtomach and inteſtinal canal as the occaſion of almoſt all their complaints.

Reaſoning in this manner, *à priori*, we ſhould expect in the cure of their diſeaſes the practice to be ſimple and the event ſucceſsful. In the adult ſtate we know that there are few morbid cauſes leſs noxious to the human frame than acidity, and few more ſubject to the controul of medicine. A little experience and reflection ſhould, in my opinion, be ſufficient to convince an unprejudiced mind that the mortality of infants is much greater than could reaſonably be expected, if the preceding theory, with regard to the exciting cauſes of their diſeaſes, were well founded.

The four following propoſitions will be found to contain the ſubſtance of the opinions of medical writers on this ſubject:

* Vide Harris de Morbis acutis Infantum.

1ſt, That human milk is a chylous fluid, and readily affected by the kind of nouriſhment which nurſes make uſe of.

2d, That it is coagulated in the ſtomach of infants, and that it is coagulable by acids, ardent ſpirits, and other known coagula.

3d, That it is very prone to run into an aceſcent or acid ſtate.

4th, That from morbid deviations towards coagulation or acidity by far the greater number of infantile diſeaſes originate, and that a variety of ſaponaceous and abſorbent remedies ought to be uſed to counteract theſe morbid cauſes.

The firſt general propoſition, viz. "that "milk is a chylous fluid, and readily affected "by food," &c., is of a nature which it is impoſſible to decide by experiment. Pure chyle is a fluid which has hitherto been collected in ſuch ſmall quantity, that its nature and properties are not yet well underſtood. It is ſaid to coagulate on expoſure to air or by ſtagnation. If ſo, I ſhall ſoon make it appear that in this particular, at leaſt, chyle differs widely from human milk.

Whether the milk of a nurſe be readily affected by the kind of food ſhe eats, or by me-

 dicines, dicines, is a queſtion of which my own obſervation does not enable me to ſpeak with deciſion. I ſhall therefore proceed to conſider the ſecond propoſition, viz. " that milk is coagu-" lated in the ſtomach of infants, and coagu-" lable by acids, ardent ſpirits," &c. This is a generally received maxim which admits of more prompt and deciſive evidence than the former, and than which there is not perhaps one in all the medical folios more erroneous. In direct oppoſition to ſuch ſentiments, it may be ſafely aſſerted that women's milk, in an healthy ſtate, contains no coagulable mucilaginous or cheeſy principle in its compoſition, or that it contains ſo little as not to admit of ſenſible proof. The late Dr. Rutty *, whoſe indefatigable induſtry and accuracy in experiment is univerſally acknowledged, in treating of the comparative quantity of curd contained in different kinds of milk, ſtates, " that woman's " milk, mixed with a quantity of runnet equal " to what coagulated cow's milk, gave of curd " very little, even not a ſixth part of what " cow's milk did." Had he taken off the

* Analyſis of milk, appended to a pamphlet on ſulphureous waters. A. D. 1762.

cream before he added the runnet, I am perſuaded he would have ſtated the quantity of curd obtained as little or none. In fact, his concluſion implies nearly what I have ſtated: he does not inform us what the quantity obtained was, but what it was not.

Profeſſor Young's concluſion, from a number of very ſatisfactory experiments, is, that human milk is not coagulated by runnets; nor do acids, whether mineral or vegetable, mixed with it in large quantity, produce any ſeparation of curd from whey, whether the milk be tepid or raiſed to the boiling point.

Doctor Ferris, whoſe Diſſertation on Milk gained the Harveian prize medal at Edinburgh in the year 1782, confirms Young's experiments on this ſubject; and I have made a great number to the ſame purpoſe in endeavouring to detect the curd of human milk, but without ſucceſs. I made uſe of all the different kinds of acids, ardent ſpirits, infuſion of infants' ſtomachs, &c., in various proportions and degrees of temperature, and I had perhaps a greater variety of milk from different women than any of the gentlemen already mentioned, and, except in one or two inſtances, never could perceive any thing like curd. In both the in-

K 2 ſtances

ſtances to which I allude there appeared, in conſequence of ſpontaneous aceſcency, a ſmall quantity of ſoft flakey matter floating in the ſerum. Ought not an appearance which does not occur above once in forty or fifty times to be conſidered as a morbid deviation from the healthy ſtandard?

We conclude that the milk of other animals contains curd, becauſe it is readily detected by a watery infuſion of the ſtomachs of ruminating, and of ſome non-ruminant animals, by acids, by ardent ſpirits, and by the juices of certain plants, and becauſe by the admixture of theſe we are enabled to collect a quantity of viſcid matter, which, when expoſed to preſſure, is well known by the name of cheeſe. But every part of this evidence is deficient in regard to human milk. Whence then is the concluſion drawn? It is a concluſion depending on one ſingle circumſtance, viz. the appearance of the fluids vomited by infants after ſucking. In deſcribing the diſeaſes of young children, authors have been in the habit of enumerating "vomiting of curdled milk" as a frequent ſymptom, and hence ſeems to have ariſen the general opinion. But ſurely ſuch deſcriptions would have been more accurate had had they been thus ſtated — "infants often "throw up quantities of a ſoft viſcid matter, "reſembling the coagula of milk, and this is "frequently mixed with a good deal of turbid "whey-like fluid."

It appears to me ſurpriſing that Dr. Young was not able to ſolve this difficulty, after he was fully convinced that no artificial means were ſufficient to ſeparate a curd from woman's milk. "Yet," ſays he, "this ſeparation takes "place ſpontaneouſly, eſpecially if it be placed "in a ſituation equal to 96° of Farenheit's "thermometer; and it is daily obſerved in "the milk which infants vomit."—It deſerves to be here remarked, that theſe obſervations are ſtated as matter of opinion, and not as the reſult of any experiment. I determined, however, to try them as far as poſſible by this teſt. I took equal quantities of three different kinds of milk, put them into bottles ſlightly corked, and theſe bottles into water, the temperature of which was kept up by a ſpirit of wine lamp as near to 96° of Farenheit as poſſible. But after frequently examining each bottle, during the courſe of the experiment, at the expiration of ſeveral hours there was not the ſmalleſt tendency towards coagulation to be perceived in any any of them. As ufual, the cream was thrown to the furface thick and adhefive, and entirely feparated from the fluid underneath, which had fomewhat of a gray wheyifh appearance.

As the matter vomited by infants is fometimes more adhefive than we might fuppofe cream to be, I fufpected that the curd might be fo entangled with the cream as to be with difficulty feparated from it; I therefore collected a quantity of rich cream from a large quantity of milk of different women, and repeated the former experiment with precifely the fame event. Towards the conclufion I added acids, both mineral and vegetable, but without producing any thing like curd. Indeed I had little doubt, before any experiment was attempted on this fubject, that Dr. Young was miftaken in the idea of milk feparating into curds and whey in a certain degree of temperature; for, was this fact, we fhould every day meet with ftagnant milk in the mammæ, where it is expofed to the heat of the human body, thus feparating and producing very troublefome obftructions; but this we know does not take place.

That the powers of an infant's ftomach may produce effects on milk which no other power can, can, is extremely poſſible; but that it cannot create any new principle, or cauſe a ſeparation of a principle which it does not contain, can hardly be doubted. Repeated experiments have ſhewn that the ſtomachs of ruminant animals, for ſome time after death, poſſeſs ſome of their moſt remarkable powers while living, and particularly that of coagulating milk: there is every reaſon to expect the ſame of the human ſtomach, and in ſeveral trials we have not been diſappointed.

I took out the ſtomach of a fœtus deprived of life in the birth by leſſening the bulk of its head. The gaſtric fluids in ſuch a ſtomach could neither be altered by diſeaſe nor the admixture of food. I infuſed it in a ſmall quantity of hot water, ſo as to make what might be conſidered a ſtrong infuſion. To equal quantities of cow's and human milk I added a tea-ſpoonful of the above infuſion: in a ſhort time the cow's milk was firmly coagulated; the human not in the leaſt changed. At the end of the firſt hour I added a ſecond tea-ſpoonful of runnet to the human milk, and ſoon after a third, without producing the ſmalleſt perceptible tendency to coagulation.

Upon the whole, then, I am perfuaded it will be found that human milk, in an healthy ftate, contains little or no curd, and that the general opinion of its nature and properties is founded on fallacious analogy and fuperficial obfervations made on the matter vomited by infants.

We may prefume that the cream of woman's milk, by its inferior fpecific gravity, will fwim on the furface of the contents of the ftomach, and being of an oily nature, that it will be of more difficult digeftion than any other conftituent part of milk. When an infant fucks very plentifully, then, fo as to over diftend the ftomach, or labours under any weaknefs in the powers of digeftion, it cannot appear unreafonable to fuppofe that the cream fhall be rejected firft by vomiting. Analogous to this we know that adults affected with dyfpepfia often bring up greafy fluids from the ftomach by eructation, and this efpecially after eating fat meat. We have in fome inftances known this to blaze when thrown into a fire, like fpirits of wine or oil.

That vifcid cream has given rife to the opinion of curd in the milk vomited by infants, is ftill farther confirmed by the following fact:—

Having

Having conftantly obferved that the milk of women, for fome days after delivery, threw up a copious yellow cream, it occurred to me, that, if my ideas on this fubject were juft, what is commonly called curds, as vomited by infants, ought to be of a yellow colour for the firft few days after birth. Accordingly I put this queftion to all our experienced nurfe-tenders in the Lying-in Hofpital — " Is there any " difference of colour in the curds vomited by " infants of four or five days old and by thofe " of a fortnight or three weeks ?"—It happened that two or three of them were fitting together when I firft thought of propofing this queftion. They anfwered unanimoufly, and without hefitation, " Surely, Sir, there is; " until the beefting milk is over the curds are " yellow, and afterwards they become white."

I fhall now haften to confider the third general propofition, viz. " that woman's milk is " prone to run into an acefcent or acid ftate." Acefcency and acidity are relative terms, and can be applied with propriety only in confequence of accurate comparifon. Whoever takes the trouble of attentively comparing human milk with that of the ruminant animals will foon find it to be much lefs prone to run into the acefcent or acid procefs. I have very often expofed equal quantities of human and cow's milk in degrees of temperature, varying from the common fummer heat, or 65°, to 100°, and I have conftantly found that cow's milk acquires a greater degree of acidity in thirty-fix hours than the human did in many days. Cow's milk becomes offenfively putrid in four or five days; a change which healthy human milk, expofed in the fame manner, will not undergo in many weeks, nay fometimes in many months. I once kept a few ounces of a nurfe's milk, delivered about fix or feven days, for more than two years in a bottle moderately corked. It ftood on my chimney piece, and was frequently open-ed to be examined. At the end of this period it fhewed evident marks of moderate acidity, whether examined by the tafte, fmell, or paper ftained by vegetable blues or purples; the lat-ter it changed to a florid red colour: whereas cow's milk, kept a few days, changed the co-lour of the fame paper to a green, thereby clearly fhewing its putrefcent tendency.

Doctor Young obferves, in general, that the milk of the whole clafs of non-ruminant ani-mals is lefs acefcent than that of the ruminant.

I have

I have been able to find but one other author whose observations at all coincide with mine, and for his authority I am indebted to the industry of the late Baron Haller. This author is a M. Navier. His words are, "*Lac femininum nullum prodit acoris signum. Post quadraginta et tres integros dies non magis acet quam lac vaccæ recens.*" Haller's observation on this passage is, "*Ea vis est victus animalis*;" and thus he seems to think this singularity accounted for. But many of my experiments were made on the milk of women rigidly confined to gruel, bread, and whey, and therefore the phænomenon observed by Navier was probably not the effect of animal diet. Perhaps another instance could not be adduced of an animal fluid resisting so powerfully the changes produced on most bodies by fermentation. Whether it is to be attributed to the saccharine nature of milk taking up a length of time in going through a vinous fermentation previous to the acid stage, or whether this saccharine principle, so abundant in human milk, be of an antiseptic nature, and thus prevents the other principles from running into the putrid stage of fermentation, I shall not pretend to determine. Of the fact I have no doubt, however it may be

 explained, explained. If we find milk out of the body ſo very ſlow in running into an aceſcent ſtate, does it not afford ſtrong preſumptive evidence that the milk of nurſes cannot be ſo very prone to run into acidity in the ſtomachs of infants as authors endeavour to perſuade us?

Our ſuſpicions on this head will be ſtrongly increaſed, if, on reviewing the ſigns ſuppoſed to indicate acid acrimony, they be found deficient and inconcluſive. Curdled milk and green four-ſmelling fæces are the marks which have been generally thought to characteriſe predominant acidity. Enough, I hope, has been already ſaid to expoſe the miſtaken notions derived from the firſt appearance, viz. "curdled milk." Againſt the ſecond we are enabled to ſpeak on the authority of Sydenham. In his letter to Dr. Cole, on hyſteria, he aſſerts, that the green herbaceous-coloured ſtuff thrown up in hyſteric cholic is no proof of acrid humours being the cauſe of the diſeaſe; for, ſays he, healthy people, when ſea-ſick, evacuate ſimilar matter. And further, let us take his own words, "*An-*

"*non et infantes in paroxyſmis convulſivis, in*

"*quibus ſpirituum animalium maxime res agitur,*

"*tam per ſuperiora quam per inferiora materiam*

"*ejuſdem plane coloris ejiciunt? Emeticis etiam*

" et

"*et catharticis frequentius propinatis uberior* ma-

"teriæ viridis *naſcitur ſeges. Et profecto,*" Says he elſewhere, "*ita lubrica eſt et evanida colorum* "*ſpeculatio ut nihil certi ex illis de corporum in* "*quibus adparent natura queat deprehendi.*"

The opinion of green fæces in infancy being occaſioned by predominant acidity, reſts very much on a ſuppoſition that bile and acid mixed produce a green compound. Sylvius ſays, "*Non* "*dubitamus aſſeverare, ortum habere notatam al-* "*vi dejectionem* virideſcentem *à bile ab acido* "*acri corruptâ, et in virorem deductâ; quales* "*mutationes colorum, haud ignotæ ſunt tinctori-* "*bus.*"

Harris, who expanded the doctrine of Sylvius, and who, from his high rank and ſuppoſed ſucceſs in practice, brought this doctrine into great faſhion in England, ſays, "*Quod viridis* "*fæcum color acido bili admixto ſe prorſus debeat,* "*obſervationi plane ſenſibili illorum, qui experiri* "*amant colorum in viridem mutationes aceto et* "*ſpiritibus acidis perficiendas, evidentiſſime appa-* "*rebit.*"—Having already found many aſſertions equally poſitive to be erroneous, I determined to doubt every thing advanced on the ſubject; I therefore procured ſome bile from the gall bladder of a fœtus which was deprived of of life in the birth: it was of a deep yellow colour and thick confiftence. After diluting fome of this bile with milk, I gradually dropped into it fome ftrong vinegar, without being able to perceive the leaft change of colour; whereas on adding nitrous acid, even in very fmall quantity, to a portion of the fame diluted bile, it immediately changed it to a deep green. This experiment I repeated in prefence of fome of the pupils of the Lying-in Hofpital with precifely the fame event. I fhould not venture to ftate two experiments as proof in any doubtful cafe, did I not find them confirmed by Dr. Maclurg, the lateft and moft accurate experimenter on this fubject. In the firft eight experiments * on human cyftic bile this author endeavours to afcertain the effects of mineral acids on bile; thefe he found, when applied ftrong, united with it and diffolved it in a fhort time, with fome variations of the phænomena, producing a fine green colour.

In the ninth and tenth experiments this author gives an account of the effects of vegeta-

* See an analyfis of Dr. Maclurg's experimental inquiry on bile in the Edinburgh Medical Commentaries, Vol. I. page 150.

ble ble acid on bile. He found that vinegar and lemon juice inſtantly coagulated it; at the ſame time changing its colour to yellow.

From theſe facts, then, it appears that the aſſertions of Sylvius, Harris, and others, in regard to the mixture of bile and acids, are but partially true. It is to be remembered that the mineral acids only form a green compound with bile. Nothing equivalent to any of the mineral acids can with probability be ſuppoſed to be generated in the inteſtines of an infant, and therefore recourſe muſt be had to ſome other mode of accounting for their green fæces. Why ſhould ſour milk, granting its exiſtence, give riſe to them in infants and not in adults? Have butter milk, ſummer fruits of the moſt aceſcent kind, lemon or orange juice, always this effect in adults by their admixture with bile? This is a queſtion which cannot, I believe, be anſwered in the affirmative.

Upon the whole, I hope it will appear probable to the generality of readers, that predominant acidity in the primæ viæ is by no means ſo general as to be conſidered the only, or even principal, ſource of infantile diſeaſes; that ſuch a morbid cauſe may now and then occur in infancy as in adult age, from weakneſs of ſtomach, mach, coſtiveneſs or improper food, can admit of no doubt. Fæces changing paper ſtained by vegetable blues and purples to a red colour, afford ſatisfactory evidence of the fact; but any concluſions drawn from their colour or ſmell muſt, from the nature of things, be liable to great uncertainty. Thoſe writers who have laid the greateſt ſtreſs on ſuch appearances in infancy do not pretend to apply the information to be derived from them to the treatment of the diſeaſes of adults.

The fourth general propoſition, viz. "that "from morbid deviations towards coagulation, "or acidity in the milk of nurſes, the greater "number of infantile diſeaſes originate, &c.," I think extremely doubtful, and for the following reaſons:

Woman's milk, in an healthy ſtate, contains little or no coagulable matter or curd.

It ſhews leſs tendency out of the body to become aceſcent than many other kinds of milk.

The appearances which have been generally ſuppoſed to characteriſe its acidity do not afford ſatisfactory evidence of the exiſtence of ſuch a morbid cauſe.

But granting ſuch acidity to prevail, we are in poſſeſſion of many harmleſs medicines (called abſorbents)

abforbents) capable of neutralifing acids, and thus forming innocent compounds. We have every advantage to be wifhed in exhibiting fuch remedies. They have no tafte; they may be fafely given in large quantities; they may be freely ufed both by the nurfe and infant to prevent as well as to cure fuch difeafe, and notwithftanding we have every day the mortification to fee infants languifh and die under fuch courfes.

The young of all the ruminant animals, fed on milk of a much more acefcent nature, fuffer no inconvenience from this fource.

Hiftory furnifhes examples of whole nations ufing four curdled milk as part of their daily food. We cannot fuppofe that fuch a practice would be continued were it often followed by pernicious effects.

Regifters of births and deaths prove, that, in one fituation, a half of the whole human race born dies under the age of two or three years; whereas in another fituation one half fhall live to the age of thirty-five or forty years and upwards.

From the fame authority it appears, that in every fituation and country a much greater proportion of the male fex dies than of the female,

 and and particularly in early infancy. In the Lying-in Hofpital of this city, during a period of about twenty-feven years, of three thoufand one hundred and ten infants dead under the age of fourteen days, one thoufand feven hundred and feventy-two were of the male fex, one thoufand three hundred and thirty-eight of the female; the deaths of the former exceeding that of the latter nearly by one third.

Such are my reafons for doubting of the prevailing opinions concerning human milk and the origin of infantile difeafes. Neither the affectation of fingularity, nor the defire of fubftituting any new theory in place of that commonly received, have had any fhare in prompting me to ftate thefe doubts to this Academy. I have been actuated to do fo folely by the hope of exciting others to inquire after truth. I do not expect that my arguments will afford conviction to any firm believer of the eftablifhed opinions on this fubject; the authority of one man is rarely fufficient to overturn or even invalidate an opinion generally and long received, efpecially when the nature of the fubject does not admit of demonftrative proof. The united labours of Willis, Baglivi, Hoffman, and Cullen, were neceffary to reform the humoral pathology thology of their predeceſſors in regard to the diſeaſes of adults; the hypotheſis of almoſt all diſeaſes being produced by morbific matter and various kinds of acrimony abounding in the human fluids ceaſes to be believed, nay is generally denied.

I cannot conclude without expreſſing a hope that a well-directed attention from phyſicians of the preſent or ſucceeding age may ſtrike out a more rational and ſuccesſful ſyſtem of practice than the preſent in regard to the diſeaſes of infancy.

